# Differential Impact of Metabolic Bariatric Surgery Versus Semaglutide on Adverse Hepatic and Extrahepatic Outcomes in Individuals With Metabolic Dysfunction‐Associated Steatotic Liver Disease and Type 2 Diabetes

**DOI:** 10.1111/dom.70757

**Published:** 2026-04-13

**Authors:** Weronika Stupalkowska, Alex E. Henney, David R. Riley, Eric G. Sheu, Uazman Alam, Daniel J. Cuthbertson

**Affiliations:** ^1^ Department of Cardiovascular and Metabolic Medicine Institute of Life Course and Medical Sciences, University of Liverpool Liverpool UK; ^2^ Laboratory for Surgical and Metabolic Research Brigham and Women's Hospital Boston Massachusetts USA; ^3^ Metabolism & Nutrition Research Group Liverpool University Hospitals NHS Foundation Trust Liverpool Merseyside UK; ^4^ Liverpool Centre for Cardiovascular Sciences University of Liverpool and Liverpool University Hospitals NHS Foundation Trust Liverpool Merseyside UK; ^5^ Division of General and GI Surgery Mass General Brigham Boston Massachusetts USA

**Keywords:** bariatric surgery, cohort study, database research, fatty liver disease, semaglutide, type 2 diabetes

## Abstract

**Aim:**

We compared the impact of metabolic bariatric surgery (MBS) versus semaglutide on clinical outcomes in individuals with metabolic dysfunction‐associated steatotic liver disease (MASLD) and type 2 diabetes (T2D).

**Methods:**

Patients with MASLD and T2D who had MBS or semaglutide in 2018–2023 were identified (TriNetX database). The primary outcome was a composite of major adverse liver outcomes (MALO): decompensation events or liver transplant. Secondary outcomes included a composite of major adverse cardiovascular events (MACE), first diagnosis of cirrhosis, heart failure, or obesity‐associated cancer (OAC), and all‐cause mortality (ACM). Subgroup analyses were performed for Roux‐en‐Y gastric bypass (RYGB) and sleeve gastrectomy (SG).

**Results:**

MBS, compared with semaglutide, was associated with a higher hazard rate (HR) of MALO (HR 1.63; 95% CI 1.12–2.37); this was driven by RYGB (2.01; 1.26–3.20) but not by SG (0.97; 0.52–1.78). However, after excluding patients with pre‐existing cirrhosis, MBS was associated with a reduced HR of new cirrhosis (0.46; 0.29–0.73). For extrahepatic events, MBS was associated with a reduced HR of MACE (0.51; 0.38–0.67), heart failure (0.41; 0.27–0.62), and OAC (0.56; 0.34–0.92). There was no difference in ACM between MBS and semaglutide, but in subgroup analysis, RYGB was associated with increased ACM compared to semaglutide (1.64; 1.01–2.67).

**Conclusion:**

In patients with MASLD and T2D, MBS, and specifically RYGB, as compared to semaglutide, may drive a relatively increased risk of MALO despite protection against MACE, the first diagnosis of cirrhosis, heart failure, and OAC. Liver evaluation in individuals with T2D referred for MBS may help optimize their treatment.

## 1 Introduction

Steatotic liver disease (SLD) describes excessive lipid accumulation in the liver, due to various causes including obesity, alcohol, drugs, etc. [1]. Metabolic dysfunction‐associated steatotic liver disease (MASLD), a condition in which SLD co‐exists with ≥ 1 cardiometabolic factor [2], accounts for over 95% of SLD cases [3] and affects nearly 40% of adults worldwide [4]. Unfortunately, the global prevalence of MASLD continues to increase [4], creating a significant burden on healthcare systems [5]. Patients with MASLD have an increased risk of adverse hepatic and extrahepatic outcomes, driven further by co‐existence of type 2 diabetes (T2D) [4, 6]. Consequently, MASLD is now the most common indication for liver transplant in the US female population [4], while cardiovascular disease remains the main cause of death in individuals with MASLD [4].

Metabolic bariatric surgery (MBS) remains superior to medical therapy in the management of obesity and T2D [7], due to the greater magnitude of weight loss and considering drug intolerance or discontinuation due to cost [8]. Furthermore, MBS has been recognized as a treatment option for individuals with MASLD (who meet the body mass index (BMI) thresholds for surgery), given the postoperative improvements in histological endpoints [9, 10]. Reflecting this, we have recently demonstrated that MBS is associated with reduced rates of hepatic and extrahepatic morbidity, and all‐cause mortality (ACM) in patients with SLD [11]. However, special consideration should be given to patients with cirrhotic MASLD, because in those patients, MBS, and especially Roux‐en‐Y gastric bypass (RYGB), is associated with a higher risk of postoperative and liver‐related complications [12].

Semaglutide is the first and only glucagon‐like peptide 1 receptor agonist (GLP‐1RA) approved in the USA for the treatment of adults with (non‐cirrhotic) metabolic dysfunction‐associated steatohepatitis (MASH) and moderate‐to‐advanced fibrosis [13], following a clinical trial reporting resolution of steatohepatitis in 63% and reduction in fibrosis in 37% of patients taking semaglutide as compared to placebo [14]. Together with resmetirom [15], a thyroid hormone receptor‐beta agonist, these two agents remain the only pharmacological options currently available to patients with advanced MASLD.

With this in mind, we compare the impact of MBS versus semaglutide on hepatic and extrahepatic outcomes and ACM in individuals living with any stage of MASLD (including cirrhotic MASLD) and T2D. The purpose of focusing our analysis specifically on individuals with co‐existing MASLD and T2D is two‐fold: first, those patients are at particularly high risk of adverse clinical outcomes [4, 6], and second, to avoid potential selection bias given that semaglutide was only approved for use in non‐diabetic patients in 2021 [16].

## 2 Methods

### 2.1 Study Design

This is a retrospective cohort study utilizing data from the Global Network on the TriNetX platform. TriNetX is a global federated health research network which holds de‐identified data supplied by the participating healthcare organizations worldwide [17]. It contains records from over 135 million patients, and the majority of the participating institutions are in North America [17]. Data extraction and analysis for this study was conducted between November 2025 and March 2026.

### 2.2 Study Population

#### 2.2.1 Inclusion

Individuals ≥ 18 years old with steatosis or steatohepatitis and T2D (Table S1).

#### 2.2.2 Exclusion

Individuals with prior liver transplant, gastric cancer, alcohol‐related disorders and those with alcohol‐associated liver disease (ALD) or other causes of chronic liver disease (Table S2). As a result, the population in this study includes individuals with pure MASLD or MASLD with increased alcohol consumption (MetALD) [2]. We also excluded patients taking weight loss medications other than semaglutide and those who had MBS other than RYGB or sleeve gastrectomy (SG). Additionally, patients in the MBS cohort could not have taken semaglutide at any point in time, and patients in the semaglutide cohort could not have had MBS of any type at any point in time.

### 2.3 Study Cohort Creation

**FIGURE 1:**
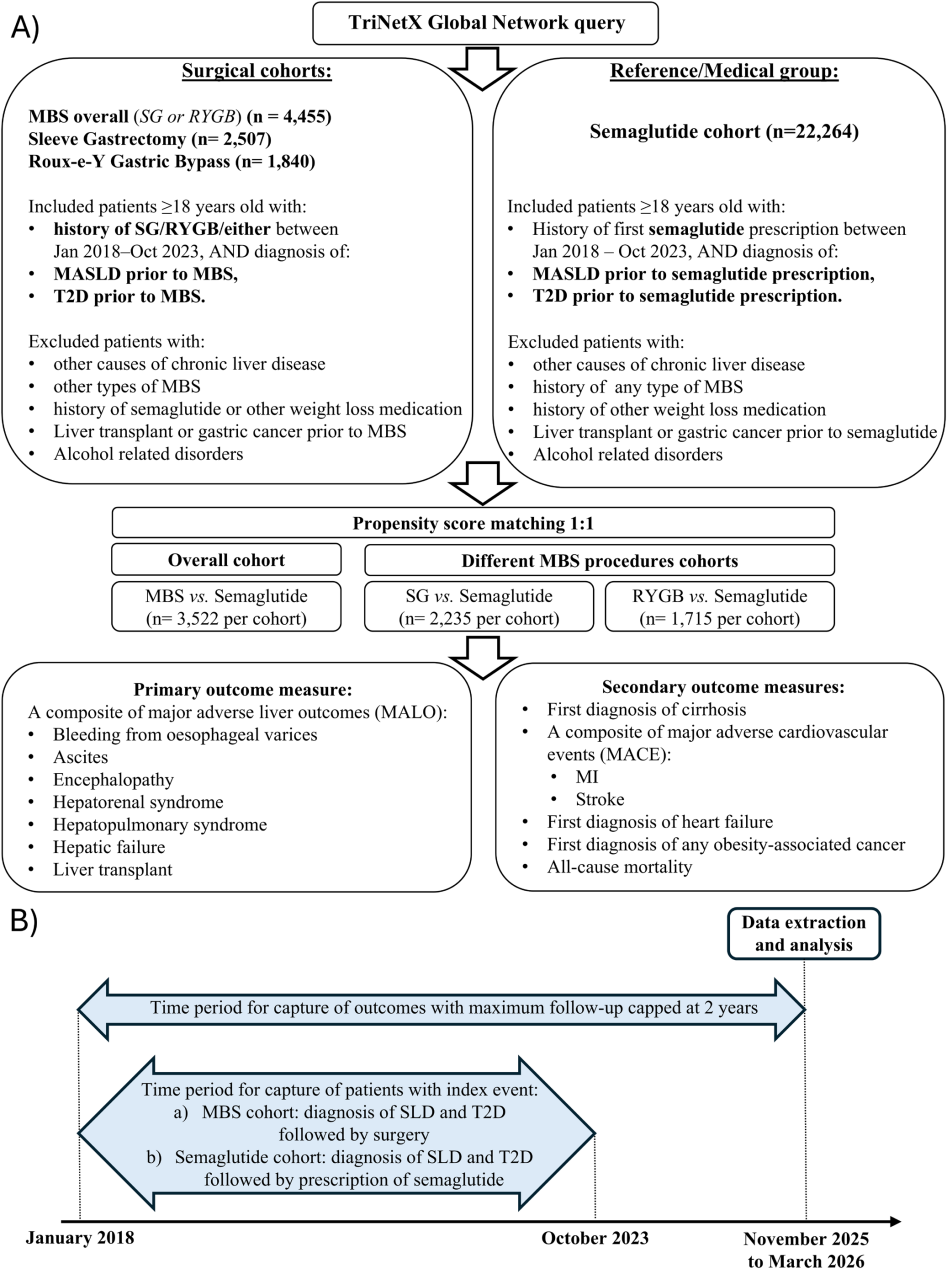
Outline of cohort construction and analysis approach (A) and study timeline (B). MACE, major adverse cardiovascular events; MALO, major adverse liver outcomes; MASLD, metabolic dysfunction‐associated steatotic liver disease; MBS, metabolic bariatric surgery; MI, myocardial infarction; RYGB, Roux‐en‐Y gastric bypass; SG, sleeve gastrectomy; T2D, type 2 diabetes.

The study population was divided into cohorts: those who underwent either SG or RYGB after their diagnosis of MASLD and T2D (MBS cohort), those who underwent SG only (SG cohort), those who underwent RYGB only (RYGB cohort), and those who were prescribed semaglutide (semaglutide cohort) after their diagnosis of MASLD and T2D. The semaglutide cohort served as the reference group (Figure 1A).

Additionally, we examined outcomes in two other MASLD subgroups: patients who did not have pre‐existing cirrhosis (i.e., non‐cirrhotic MASLD) and separately patients with MASLD but without T2D. In the latter analysis, the time window for subject capture is limited to 2021–2023, that is, after semaglutide was approved for weight loss [16], and therefore could have been prescribed to nondiabetic individuals.

### 2.4 Index Event

The index event in surgical cohorts was defined as the date of surgery. The index event in the semaglutide cohort was defined as the date of first semaglutide prescription. The index event must have occurred between January 2018 (the first year after introduction of semaglutide into clinical practice) and October 2023 (to allow a 2‐year follow‐up for all individuals, regardless of the time they were recruited) (Figure 1B).

### 2.5 Follow‐Up

To counteract the potential survival bias associated with time‐to‐treatment initiation in the semaglutide group [18], we defined the start of follow‐up as 60 days after the index event. We also performed a sensitivity analysis where the start of follow‐up is set to 90 days after the index event to account not only for delays in treatment initiation but also dose titration. The end of follow‐up was set to 2 years after the index event. Individuals' follow‐up ended either at the first occurrence of the outcome of interest or after they reached the end of the follow‐up window. Individuals without the outcome were censored either at the end of follow‐up or after the date of their last known medical record.

### 2.6 Primary and Secondary Outcome Measures

*The primary outcome* was a composite of major adverse liver outcomes (MALO), defined as: bleeding from oesophageal varices, ascites, encephalopathy, hepatorenal or hepatopulmonary syndrome, hepatic failure or liver transplant [19] (Table S3).

*The secondary outcomes* included: first diagnosis of liver cirrhosis, major adverse cardiovascular events (MACE; a composite of acute myocardial infarction and stroke), first diagnosis of heart failure, first diagnosis of any obesity‐associated cancer [20], and ACM.

### 2.7 Definitions of ‘First Ever’ Versus ‘Any’ Diagnosis

For MALO and MACE (i.e., outcomes that can recur/relapse) we analysed the cohorts looking either at the *first ever diagnosis* (labelled as ‘MALO (*first*)’ and MACE (*first*) respectively) or at *any* diagnosis (labelled as ‘MALO (*any*)’ and ‘MACE (*any*)’, respectively). In case of *first* diagnosis, the ‘at risk population’ excluded individuals with the respective outcome occurring before the start of follow‐up window. In case of *any* diagnosis, the ‘at risk population’ included both outcome‐ naïve individuals and those who experienced the relevant outcome in the past.

For cirrhosis, heart failure and obesity‐associated cancer we analysed the cohorts looking at *first ever diagnosis* only, that is, individuals with the respective outcomes occurring before the start of follow‐up window were excluded from the ‘at risk population’.

### 2.8 Propensity Score Matching

To balance the cohorts' baseline characteristics, propensity score matching (PSM) in a 1:1 ratio was applied. The covariates used in the PSM include age, sex, race, BMI, haemoglobin A1C (HbA1C) and established risk factors for MASLD [21] and cardiovascular disease [22] (Table S4). Additionally, to match for the severity of MASLD, we included codes for hepatic fibrosis and cirrhosis. BMI and HbA1C were treated as categorical variables in the PSM. The stratification thresholds for these variables were selected based on common reference values [2, 23, 24, 25, 26] and adjusted accordingly to achieve satisfactory balance in the cohorts' mean values.

### 2.9 Statistical Analysis and Data Visualization

Continuous data are presented as means ± standard deviation (SD) and discrete data are presented as counts and percentages. PSM was performed using logistic regression and greedy nearest‐neighbour matching with a calliper of 0.1 pooled SD and difference between propensity scores of less than 0.1. The standardized mean difference (SMD) was used to assess covariate balance between the cohorts with a threshold of less than 0.1 for the cohorts to be considered well‐balanced. Finally, survival analysis and log‐rank test were performed to generate Kaplan–Meier curves and hazard ratios (HRs), which are presented with 95% confidence intervals (CI) and *p*‐values for hazard proportionality test. If *p* value for the latter was found to be ≤ 0.05 and the HR was significant, it was interpreted as a violation of the proportionality assumption. This occurred only in the no T2D subgroup analysis for the MALO (*first*) outcome; however, the HR was not significant, therefore no further analysis was undertaken.

Data visualization was performed using GraphPad Prism (v10.3.0) and forestplot package [27] (v3.1.7) within R Environment [28] (v4.5.2).

## 3 Results

### 3.1 Study Cohorts

Between 2018 and 2023, we identified a total of 4455 patients with MASLD, T2D, and a history of MBS (MBS cohort), 2507 individuals with a history of SG (SG cohort), 1840 individuals with a history of RYGB (RYGB cohort), and 22 264 individuals with MASLD and T2D who received their first prescription of semaglutide during this time window (semaglutide cohort) (Figure 1A). After PSM, the cohort size was reduced to 3522 patients per group in the primary analysis (MBS vs. semaglutide); 2235 and 1715 individuals per cohort in the SG and RYGB subgroup analyses, respectively (Table 1 and Tables S5 and S6). Additionally, when assessing the *first‐ever* outcome occurrence, the cohort sizes were reduced further by excluding patients in whom the respective outcome of interest occurred before the start of the follow‐up window (Table 2).

**TABLE 1:** Baseline characteristics of patients with co‐existing MASLD and T2D, with history of metabolic bariatric surgery or semaglutide use in 2018–2023.

Characteristic	Before PSM			After PSM		
	MBS *n* = 4455	Semaglutide *n* = 22 264	SMD	MBS *n* = 3522	Semaglutide *n* = 3522	SMD
Age, mean [SD], years	47 [12]	58 [13]	0.882	49 [12]	49 [13]	0.010
Female sex, *n* (%)	3201 (71.9)	13 084 (58.8)	0.278	2454 (69.7)	2428 (68.9)	0.016
Race and ethnicity, *n* (%)						
Asian	52 (1.2)	1297 (5.8)	0.256	50 (1.4)	57 (1.6)	0.016
Black or African American	657 (14.7)	2483 (11.2)	0.107	492 (14.0)	485 (13.8)	0.006
Hispanic or Latino	801 (18.0)	1836 (8.2)	0.291	547 (15.5)	514 (14.6)	0.026
White	2865 (64.3)	16 402 (73.7)	0.203	2335 (66.3)	2311 (65.6)	0.014
Anthropometric and biochemical measurements, mean [SD]						
BMI, kg/m^2^	44.8 [8.2]	36.7 [7.6]	1.016	43.5 [8.0]	42.7 [7.8]	0.096
HbA_1C_, *%*	6.9 [1.3]	8.2 [1.8]	0.835	7.1 [1.3]	7.2 [1.5]	0.069
Comorbidities, *n* (%)						
Hepatic Fibrosis	43 (1.0)	167 (0.8)	0.023	38 (1.1)	34 (1.0)	0.011
Hepatic Cirrhosis	51 (1.1)	573 (2.6)	0.106	48 (1.4)	48 (1.4)	< 0.001
Hypertension	2583 (58.0)	10 045 (45.1)	0.260	1948 (55.3)	2019 (57.3)	0.041
Dyslipidaemia	1825 (41.0)	8930 (40.1)	0.017	1445 (41.0)	1471 (41.8)	0.015
IHD	272 (6.1)	2224 (10.0)	0.143	243 (6.9)	275 (7.8)	0.035
Nicotine dependence	167 (3.7)	943 (4.2)	0.025	138 (3.9)	131 (3.7)	0.010
Medication, *n* (%)						
Insulin	338 (7.6)	3576 (16.1)	0.265	318 (9.0)	368 (10.4)	0.048
Metformin	504 (11.3)	4814 (21.6)	0.281	450 (12.8)	473 (13.4)	0.019

Abbreviations: BMI, body mass index; HbA_1C_, haemoglobin A_1C_; HDL, high‐density lipoprotein; IHD, ischemic heart disease; MBS, metabolic bariatric surgery; *n*, number; PSM, propensity score matching; SBP, systolic blood pressure; SD, standard deviation; SMD, standardized mean difference; T2D, type 2 diabetes.

**TABLE 2:** Major adverse hepatic and extrahepatic clinical outcomes and all‐cause mortality in individuals with co‐existing MASLD and T2D, having had MBS or semaglutide between 2018 and 2023.

Outcome	Patients in MBS cohort, *n*	Patients with outcome in MBS cohort, *n* (%)	Patients in semaglutide cohort, *n*	Patients with outcome in semaglutide cohort, *n* (%)	Hazard ratio (CI)	*p*‐value for hazard proportionality test^c^
Hepatic outcomes						
MALO *(any)* ^a^	3522	68 (1.9)	3522	46 (1.3)	1.63 (1.12–2.37)	0.538
MALO *(first)* ^b^	3398	45 (1.3)	3401	28 (0.8)	1.78 (1.11–2.86)	0.242
Cirrhosis *(first)* ^b^	3383	25 (0.7)	3383	60 (1.8)	0.46 (0.29–0.73)	0.763
Extrahepatic outcomes						
MACE *(any)* ^a^	3522	73 (2.1)	3522	156 (4.4)	0.51 (0.38–0.67)	0.404
MACE *(first)* ^b^	3320	39 (1.2)	3221	76 (2.4)	0.55 (0.37–0.81)	0.108
Heart failure *(first)* ^b^	3214	31 (1.0)	3004	78 (2.6)	0.41 (0.27–0.62)	0.160
Obesity‐associated cancer *(first)* ^b^	3319	24 (0.7)	3229	46 (1.4)	0.56 (0.34–0.92)	0.197
All‐cause mortality						
Deceased	3522	51 (1.4)	3522	55 (1.6)	1.03 (0.70–1.50)	0.648

Abbreviations: CI, confidence interval; MACE, major adverse cardiovascular events; MALO, major adverse liver outcomes; MBS, metabolic bariatric surgery; *n*, number; obesity‐associated cancer is defined as cancer of any of the following: colon, sigmoid, rectum, pancreas, gallbladder, stomach, oesophagus, hepatocellular carcinoma, breast, endometrium, ovary, kidney, thyroid, meninges, and multiple myeloma.

^a^ Any diagnosis includes outcomes recorded during the study follow‐up that are either first ever occurrence or a recurrence, that is, the outcome could have occurred in the past before the subject recruitment into the study or before the start of follow‐up.

^b^ First diagnosis signifies first ever occurrence only, that is, no previous history of a given outcome before the subject recruitment into the study and start of follow‐up.

^c^ In the hazards proportionality test, if the *p* value is > 0.05, the hazard ratio remains constant over time.

### 3.2 Baseline Characteristics

After PSM, the mean age was 49 ± 12 and 49 ± 13 years, and female patients comprised 69.7% and 68.9% of the MBS and semaglutide cohorts respectively (Table 1). Overall, the cohorts were considered well‐matched. After PSM, the mean follow‐up was 20.8 ± 7.2 (MBS) and 22.9 ± 4.6 (semaglutide) months. Baseline characteristics of patients in the SG, RYGB, *non‐cirrhotic* MASLD and no‐T2D cohorts are presented in Tables S5–S8. Notably, for BMI and HbA1C, the groups were matched according to subgroup categories rather than the mean values. Whilst in the main analysis (MBS vs. Semaglutide) an adequate balance in the mean values of BMI and HbA1C was achieved between the cohorts (mean BMI in MBS vs Semaglutide: 43.5 ± 8.0 vs 42.7 ± 7.8, SMD = 0.096; mean HbA1c: 7.1 ± 1.3 vs 7.2 ± 1.5, SMD = 0.069), in the SG and RYGB analyses, there were small differences in the mean value of HbA1c between the surgical cohorts and the semaglutide group (SG vs. Semaglutide: 7.0 ± 1.4 vs. 7.1 ± 1.4, SMD = 0.104 and RYGB vs. Semaglutide: 7.0 ± 1.3 vs. 7.1 ± 1.4, SMD = 0.109, Tables S5 and S6). Additionally, in the SG vs. Semaglutide analysis, there was also a small difference in the mean BMI, favouring the semaglutide group (45.0 ± 7.6 vs. 43.6 ± 8.1, SMD = 0.176, Table S5). Finally, in the analysis of patients with non‐cirrhotic MASLD, there was again a small imbalance between the mean value of HbA1c between the MBS and semaglutide groups (7.1 ± 1.3 vs. 7.2 ± 1.5, SMD = 0.116, Table S7).

### 3.3 Hepatic Outcomes

#### 3.3.1 MALO

**FIGURE 2:**
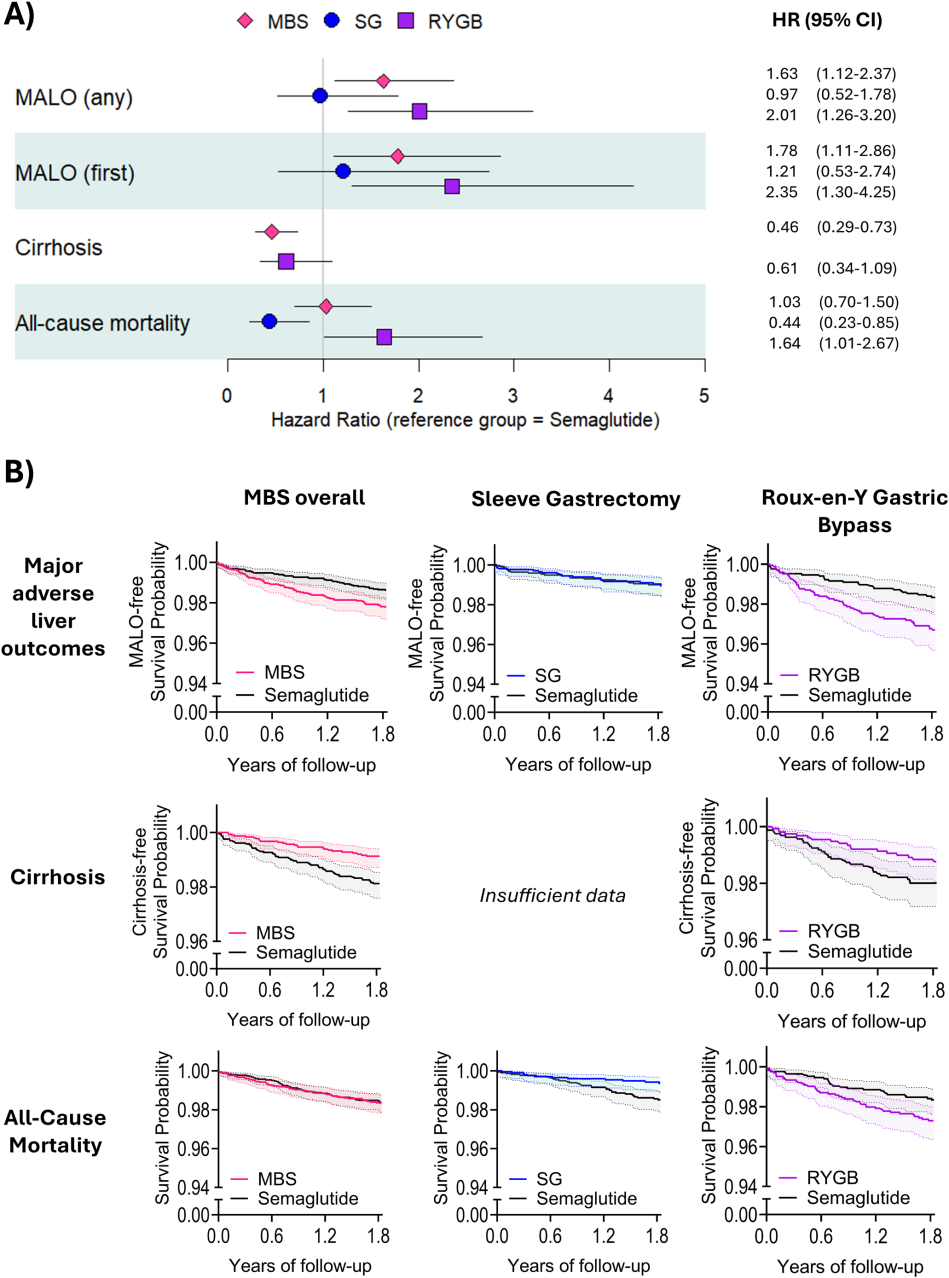
Impact of MBS versus semaglutide on adverse hepatic events and all‐cause mortality in individuals with MASLD and T2D. (A) Hazard ratios of adverse hepatic events and all‐cause mortality in primary analysis and subgroup analyses by procedure type. (B) Event‐free survival probabilities in primary analysis and subgroup analyses by procedure type. CI, confidence interval; HR, hazard ratio; MALO, major adverse liver outcomes; MBS, metabolic bariatric surgery; RYGB, Roux‐en‐Y gastric bypass; SG, sleeve gastrectomy.

Compared to semaglutide, MBS was associated with increased HR of both *any* and *first* diagnosis of MALO in patients with MASLD and T2D (HR of *any* diagnosis: 1.63, 95% CI: 1.12–2.37; HR of *first* diagnosis: 1.78, 1.11–2.86; Table 2 and Figure 2). This trend persisted after delaying the onset of follow‐up by 90 days to account for semaglutide titration (HR 1.59, 1.09–2.32, Table S9) and even after excluding patients diagnosed with liver cirrhosis prior to MBS or semaglutide prescription (HR 1.69, 1.11–2.57, Table S10). In subgroup analysis, the increased HR of MALO was associated only with RYGB and not with the SG procedure (HR 2.01, 1.26–3.20 in RYGB; HR 0.97, 0.52–1.78 in SG; Tables S11 and S12 and Figure 2). Finally, in nondiabetic patients with MASLD, there was no significant difference in the risk of MALO between the MBS and the semaglutide cohorts (Table S13) with event rates lower in both cohorts as compared to patients with T2D.

#### 3.3.2 Cirrhosis

After excluding patients with pre‐existing cirrhosis, compared to semaglutide, MBS was associated with reduced HR of *first* diagnosis of cirrhosis both in main analysis (HR 0.46, 0.29–0.73; Table 2 and Figure 2) and after delaying the onset of follow‐up by 90 days (HR 0.55, 0.35–0.88, Table S9). This trend was not significant in the remaining subgroup analyses, likely due to overall low event rate and short follow‐up time (Tables S10–S13).

### 3.4 Extrahepatic Outcomes

#### 3.4.1 MACE

**FIGURE 3:**
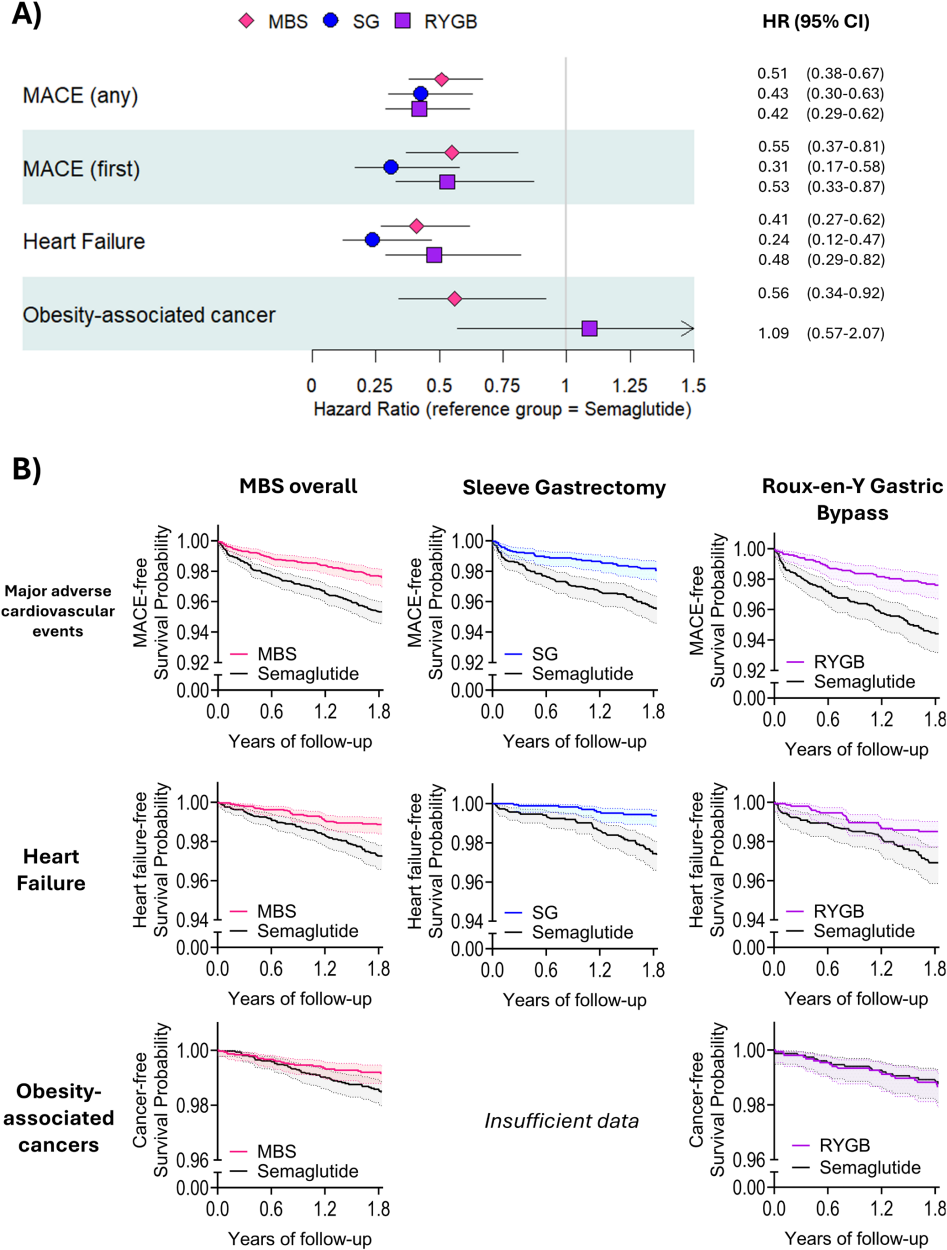
Impact of MBS versus semaglutide on adverse extrahepatic events in individuals with MASLD and T2D. (A) Hazard ratios of adverse extrahepatic events in primary analysis and subgroup analyses by procedure type. (B) Event‐free survival probabilities in primary analysis and subgroup analyses by procedure type. CI, confidence interval; HR, hazard ratio; MACE, major adverse cardiovascular events; MBS, metabolic bariatric surgery; RYGB, Roux‐en‐Y gastric bypass; SG, sleeve gastrectomy.

Compared to semaglutide, MBS was associated with reduced HR of MACE, regardless of whether it was *any* diagnosis of MACE or *first* ever MACE (HR of *any* diagnosis: 0.51, 0.38–0.67; HR of *first* diagnosis: 0.55, 0.37–0.81; Table 2 and Figure 3). The cardiovascular benefits associated with MBS were similar after delaying follow‐up by 90 days (HR 0.53, 0.40–0.70, Table S9) or after excluding individuals with cirrhosis (HR 0.48, 0.36–0.65, Table S10). In the subgroup analysis, both SG and RYGB were associated with cardiovascular health as compared to semaglutide (HR in SG of *any* MACE: 0.43, 0.30–0.63; HR of *first* MACE: 0.31, 0.17–0.58; HR in RYGB of *any* MACE: 0.42, 0.29–0.62; HR of *first* MACE: 0.53, 0.33–0.87, Tables S11 and S12 and Figure 3). Finally, in patients with MASLD but without T2D, there was no significant difference between the MBS and the semaglutide cohorts (Table S13) with overall lower event rates in both groups as compared to patients with co‐existing T2D and broad CIs indicating low precision of the estimate.

#### 3.4.2 Heart failure

MBS, as compared to semaglutide, was uniformly associated with decreased HR of *first* diagnosis of heart failure in the main analysis (HR 0.41, 0.27–0.62, Table 2 and Figure 3), after delaying follow‐up by 90 days (HR 0.44, 0.29–0.67, Table S9), after excluding patients with cirrhosis at baseline (HR 0.49, 0.30–0.80, Table S10) and with both procedure types (HR in RYGB: 0.48, 0.29–0.82; HR in SG: 0.24, 0.12–0.47, Tables S11 and S12 and Figure 3). No statistical analysis was possible in the no‐T2D cohort due to low event rate in both groups (Table S13).

#### 3.4.3 Obesity‐Associated Cancer

MBS, as compared to semaglutide, was associated with decreased HR of *first* diagnosis of any obesity‐associated cancer in patients with MASLD of any stage (HR 0.56, 0.34–0.92, Table 2 and Figure 3) and in those without cirrhosis at baseline (HR 0.59, 0.35–0.99, Table S10). The trend persisted after delaying the onset of follow‐up by 90 days (HR 0.54, 0.33–0.88, Table S9). In subgroup analyses, there were no significant differences (Tables S11–S13).

#### 3.4.4 All‐Cause Mortality

There was no significant difference in ACM between the MBS and semaglutide cohorts either in patients with MASLD of any stage (HR 1.03, 0.70–1.50; Table 2 and Figure 2) or in those without cirrhosis at baseline (HR 0.99, 0.66–1.49, Table S10). This trend persisted after delaying the onset of follow‐up by 90 days (HR 1.09, 0.74–1.61, Table S9). However, there was a differential effect on ACM depending on the MBS type. As compared to semaglutide, SG was associated with decreased ACM (HR 0.44, 0.23–0.85, Table S12 and Figure 2), whereas RYGB was associated with increased ACM (HR 1.64; 1.01–2.67, Table S11 and Figure 2).

## 4 Discussion

In this large retrospective analysis of real‐world healthcare data, we found a differential effect of MBS, as compared to semaglutide, on hepatic and extrahepatic outcomes in individuals living with co‐existing MASLD and T2D. Specifically, we noted that MBS, driven by RYGB, was associated with an increased risk of MALO in patients with established MASLD of any stage (i.e., including cirrhotic MASLD) and in patients with non‐cirrhotic MASLD (i.e., excluding individuals with liver cirrhosis at baseline). However, the risk of *de novo* diagnosis of cirrhosis after MBS was reduced as compared to semaglutide. In terms of extrahepatic outcomes, MBS, as compared to semaglutide, was associated with reduced rates of MACE, *first* diagnosis of heart failure and *first* diagnosis of any obesity‐associated cancer.

### 4.1 Impact of MBS vs. Semaglutide on Hepatic Outcomes

Direct comparisons of MBS and semaglutide effects in individuals living with MASLD are lacking, but retrospective cohort studies have shown independently that both MBS [11] and GLP‐1RAs [29] are associated with reduced risk of adverse hepatic outcomes in patients living with MASLD as compared to no treatment.

The risk of adverse hepatic outcomes rises with increasing MASLD severity, with those with liver cirrhosis having the greatest risk of MALO and ACM [30]. We therefore hypothesize that the MALO events captured in our study are predominantly recorded in individuals with advanced and cirrhotic MASLD. Unfortunately, further subgroup analysis of MALO outcomes focusing solely on individuals with cirrhotic MASLD was not feasible within the TriNetX platform due to low patient counts, reflecting the known issue of low sensitivity of ICD‐10‐CM codes for cirrhosis [31] and underdiagnosis of this condition [32]. To address this issue, we analysed outcomes in individuals with MASLD but *without* cirrhosis at baseline. Interestingly, we found the same trend in this population—MBS was still associated with higher rates of MALO as compared to semaglutide. We believe that this result must be interpreted with caution: it is possible that we have captured MALO events occurring in individuals with undiagnosed cirrhosis in this subgroup analysis.

Compared to no treatment, MBS in cirrhotic patients is associated with reduced HR of liver decompensation and MALO [33], but on the other hand, those individuals are considered to have a higher risk of postoperative complications [12]. GLP‐1RA, as compared to no treatment, has also been associated with lower risk of MALO in individuals living with co‐existent T2D and liver cirrhosis [34] and could therefore represent an alternative to surgical management of obesity and T2D in individuals with cirrhotic MASLD. Our data further supports this approach.

In subgroup analysis, only RYGB and not SG was associated with increased HR of MALO and ACM as compared to semaglutide. Furthermore, in patients with MASLD but without T2D, there was no significant difference between the effect of MBS versus semaglutide. These data suggest that if MBS is being considered in individuals with advanced MASLD and T2D, SG might be the preferred option over RYGB, which is in line with existing clinical recommendations [9]. Conversely, in those without T2D, both surgical and pharmacological options are viable.

The reason why semaglutide is superior specifically to RYGB but not SG, considering hepatic outcomes, is unclear. Historically, severe malnutrition complicating certain bariatric procedures has been associated with rare cases of hepatic failure requiring liver transplant [35]. Even though the MBS techniques have since evolved to improve the safety profile, postoperative malabsorption leading to malnutrition remains a recognized risk and occurs more commonly after RYGB than SG [36]. Moreover, RYGB is associated with developing *de novo* alcohol use disorder (AUD) which could contribute to the increased number of MALO events in those patients [37]. Other possible causes for progression of liver disease specifically after RYGB include impairment of intestinal barrier function and bacterial overgrowth in the excluded jejunal segment, leading to increased inflammation and toxin delivery to the liver [38]. These potential explanations are speculative. Finally, we cannot exclude a possibility of selection bias due to lack of specificity of the ICD10 codes relating to RYGB—in theory our analysis could have included patients who received RYGB not as a bariatric but palliative procedure due to intraabdominal malignancy—driving the increased event rate of MALO and ACM in the RYGB group.

Importantly, when we excluded patients with pre‐existing liver cirrhosis from the dataset, MBS was associated with a lower HR of *first* diagnosis of cirrhosis as compared to semaglutide. This might suggest that in the non‐cirrhotic stage of MASLD, MBS has a greater impact than semaglutide at halting disease progression, whilst offering the same cardiovascular benefits and reduced risk of obesity‐associated cancers.

Our data suggest that in terms of adverse hepatic outcomes, in patients with advanced stages of MASLD and co‐existing T2D who are at greater risk of MALO, semaglutide may offer a better therapeutic option than MBS and particularly RYGB. Conversely, in those with non‐cirrhotic MASLD, MBS confers greater benefit than semaglutide, reducing disease progression to cirrhosis. Overall, however, further research with longer follow‐up is urgently needed to allow more accurate comparisons between MBS and semaglutide in patients stratified according to MASLD severity.

### 4.2 Impact of MBS vs. Semaglutide on Extrahepatic Outcomes

Cardiovascular disease remains the main cause of death in patients with MASLD [4], therefore our finding that MBS is associated with significantly lower risk of MACE and *first* diagnosis of heart failure as compared to semaglutide is worth highlighting. It is consistent with results obtained by other authors in similar patient populations with other GLP1‐RAs [39, 40]. Furthermore, it is worth mentioning that even with a relatively short follow‐up of nearly 2 years, we demonstrate reduced HR of obesity‐associated cancer with MBS as compared to semaglutide.

Altogether, our data suggest that MBS is superior to semaglutide in terms of cardiovascular health benefits and risk reduction of obesity‐associated cancer. This, however, was not associated with a significant difference in ACM.

### 4.3 Limitations

This study has several limitations. First, due to its observational nature, no conclusions regarding causality can be drawn. Second, reliance on electronic health records is prone to confounding due to either lack or incompleteness of data on certain covariates, such as socioeconomic status and insurance. Third, errors or omissions in coding can affect both identification of individuals with MASLD as well as detection of their clinical outcomes. Fourth, the ICD‐10 codes for surgical procedures are based on anatomical descriptions rather than clinical indications and therefore might not be specific enough to exclude non‐bariatric patients from our analysis. Fifth, hepatic fibrosis and cirrhosis have relatively high rates of underdiagnosis [32] and even though we attempted to match our cohorts using codes for fibrosis and cirrhosis, it is possible that individuals with undetected/uncoded fibrosis and cirrhosis exist in our dataset and are unequally distributed across the cohorts. Sixth, in the case of pharmacological treatments (such as semaglutide), data from electronic health care databases, which rely on prescription records, might overestimate real‐life compliance. This represents a significant limitation in this study, as it is possible that our analysis contains data from patients who might have prematurely discontinued their semaglutide treatment or have never even started it. Moreover, we do not have data on individual‐level semaglutide doses, which are likely to vary depending on specific clinical indications, tolerance and time window (with lower doses used pre‐2021 for T2D treatment and higher doses in weight management afterwards). Furthermore, lack of data on semaglutide prescription filling could have introduced immortal time bias [18] which we attempted to account for by starting the follow‐up 60 and 90 days after the index event. Finally, due to lack of individual‐level data in TriNetX, we cannot stratify patients according to the severity of their MASLD.

## 5 Conclusion

Our results might suggest that MBS, as compared to semaglutide, could be associated with better cardiovascular benefits and reduced obesity‐associated cancer risk in patients with co‐existing MASLD and T2D. However, the decision on treatment options for liver health in this cohort is more nuanced. In non‐cirrhotic MASLD, MBS is associated with reduced risk of progression to cirrhosis. In contrast, in patients with MASLD of any stage (i.e., including pre‐existing cirrhosis), MBS, possibly driven by RYGB, might be associated with increased risk of MALO when compared to semaglutide. This suggests that preoperative robust liver parenchymal evaluation to screen for undiagnosed advanced fibrosis/cirrhosis and to guide weight loss management may be appropriate. These results, however, must be interpreted with caution, given the limitations of this retrospective, observational study which relies on medical coding. Further prospective research with longer follow‐up is urgently warranted.

## Funding

The authors have nothing to report.

## Conflicts of Interest

The authors declare no conflicts of interest.

## Supporting information


**Table S1:** Inclusion criteria in this study.
**Table S2:** Exclusion criteria in this study.
**Table S3:** Study clinical outcomes with associated codes.
**Table S4:** Variables included in propensity score matching in this study.
**Table S5:** Baseline characteristics of patients with co‐existing MASLD and T2D, with a history of sleeve gastrectomy or semaglutide use during 2018–2023.
**Table S6:** Baseline characteristics of patients with co‐existing MASLD and T2D, with a history of Roux‐en‐Y gastric bypass or semaglutide use during 2018–2023.
**Table S7:** Baseline characteristics of patients with MASLD and without cirrhosis, with a history of metabolic bariatric surgery or semaglutide use during 2018–2023.
**Table S8:** Baseline characteristics of patients with MASLD and without T2D, with a history of metabolic bariatric surgery or semaglutide use during 2021–2023.
**Table S9:** Major adverse hepatic and extrahepatic clinical outcomes and all‐cause mortality in individuals with MASLD and T2D, having had MBS or semaglutide between 2018 and 2023; Sensitivity analysis with follow‐up window starting 90 days after the index event.
**Table S10:** Major adverse hepatic and extrahepatic clinical outcomes and all‐cause mortality in individuals with MASLD and without cirrhosis, having had MBS or semaglutide between 2018 and 2023.
**Table S11:** Major adverse hepatic and extrahepatic clinical outcomes and all‐cause mortality in individuals with co‐existing MASLD and T2D, having had Roux‐en‐Y gastric bypass or semaglutide between 2018 and 2023.
**Table S12:** Major adverse hepatic and extrahepatic clinical outcomes and all‐cause mortality in individuals with co‐existing MASLD and T2D, having had sleeve gastrectomy or semaglutide between 2018 and 2023.
**Table S13:** Major adverse hepatic and extrahepatic clinical outcomes and all‐cause mortality in individuals with MASLD and without T2D, having had MBS or semaglutide between 2021 and 2023.

## Data Availability

Data used in this study was collected solely from the TriNetX network (https://trinetx.com). These data are not available publicly due to privacy restrictions in place. However, accredited researchers registered with TriNetX might request permission to access data via TriNetX. This may require a data‐sharing agreement and may incur data access fees.
