# Supplementary material for: Differential Impact of Metabolic Bariatric Surgery Versus Semaglutide on Adverse Hepatic and Extrahepatic Outcomes in Individuals With Metabolic Dysfunction‐Associated Steatotic Liver Disease and Type 2 Diabetes
Source: Diabetes Obes Metab. 2026 Apr 13;28(7):5684–95. doi: 10.1111/dom.70757 (PMC13243962; doi:10.1111/dom.70757)
Supplement: Supplementary file 1 — Table S1: Inclusion criteria in this study. Table S2: Exclusion criteria in this study. Table S3: Study clinical outcomes with associated codes. Table S4: Variables included in propensity score matching in this study. Table S5: Baseline characteristics of patients with co‐existing MASLD and T2D, with a history of sleeve gastrectomy or semaglutide use during 2018–2023. Table S6: Baseline characteristics of patients with co‐existing MASLD and T2D, with a history of Roux‐en‐Y gastric bypass or semaglutide use during 2018–2023. Table S7: Baseline characteristics of patients with MASLD and without cirrhosis, with a history of metabolic bariatric surgery or semaglutide use during 2018–2023. Table S8: Baseline characteristics of patients with MASLD and without T2D, with a history of metabolic bariatric surgery or semaglutide use during 2021–2023. Table S9: Major adverse hepatic and extrahepatic clinical outcomes and all‐cause mortality in individuals with MASLD and T2D, having had MBS or semaglutide between 2018 and 2023; Sensitivity analysis with follow‐up window starting 90 days after the index event. Table S10: Major adverse hepatic and extrahepatic clinical outcomes and all‐cause mortality in individuals with MASLD and without cirrhosis, having had MBS or semaglutide between 2018 and 2023. Table S11: Major adverse hepatic and extrahepatic clinical outcomes and all‐cause mortality in individuals with co‐existing MASLD and T2D, having had Roux‐en‐Y gastric bypass or semaglutide between 2018 and 2023. Table S12: Major adverse hepatic and extrahepatic clinical outcomes and all‐cause mortality in individuals with co‐existing MASLD and T2D, having had sleeve gastrectomy or semaglutide between 2018 and 2023. Table S13: Major adverse hepatic and extrahepatic clinical outcomes and all‐cause mortality in individuals with MASLD and without T2D, having had MBS or semaglutide between 2021 and 2023. [file DOM-28-5684-s001.docx]

**Supplement**

**Differential impact of metabolic bariatric surgery versus semaglutide on adverse hepatic and extrahepatic outcomes in individuals with metabolic dysfunction-associated steatotic liver disease and type 2 diabetes.**

Weronika Stupalkowska; Alex E Henney; David Riley; Eric G Sheu, Uazman Alam,
Daniel J Cuthbertson

| Contents | Page No | |
| --- | --- | --- |
| Table S1 Inclusion criteria in this study. | | **2** |
| Table S2 Exclusion criteria in this study. | | **3** |
| Table S3 Study clinical outcomes with associated codes. | | **5** |
| Table S4 Variables included in propensity score matching in this study. | | **6** |
| Table S5 Baseline characteristics of patients with co-existing MASLD and T2D,  with a history of sleeve gastrectomy or semaglutide use during 2018-2023. | | **7** |
| Table S6 Baseline characteristics of patients with co-existing MASLD and T2D,  with a history of Roux-en-Y gastric bypass or semaglutide use during 2018-2023. | | **10** |
| Table S7 Baseline characteristics of patients with MASLD and *without* cirrhosis,  with a history of metabolic bariatric surgery or semaglutide use during 2018-2023. | | **13** |
| Table S8 Baseline characteristics of patients with MASLD and *without* T2D,  with a history of metabolic bariatric surgery or semaglutide use during 2021-2023. | |  |
| Table S9 Major adverse hepatic and extrahepatic clinical outcomes and  all-cause mortality in individuals with MASLD and T2D, having had MBS or semaglutide between 2018-2023; *Sensitivity analysis with follow-up window starting 90 days after the index event.* | | **1** |
| Table S10 Major adverse hepatic and extrahepatic clinical outcomes and  all-cause mortality in individuals with MASLD and *without* cirrhosis, having had MBS or semaglutide between 2018-2023. | | **16** |
| Table S11 Major adverse hepatic and extrahepatic clinical outcomes and  all-cause mortality in individuals with co-existing MASLD and T2D, having had Roux-en-Y gastric bypass or semaglutide between 2018-2023. | | **17** |
| Table S12 Major adverse hepatic and extrahepatic clinical outcomes and all-cause mortality in individuals with co-existing MASLD and T2D, having had sleeve gastrectomy or semaglutide between 2018-2023. | |  |
| Table S13 Major adverse hepatic and extrahepatic clinical outcomes and  all-cause mortality in individuals with MASLD and *without* T2D, having had MBS or semaglutide between 2021-2023. | |  |

**Table S1** Inclusion criteria in this study.

| **Cohort** | **Code** | **Code Type** | **Description** |
| --- | --- | --- | --- |
| Both | E11 | ICD10CM | Type 2 diabetes mellitus |
|  | K76.0 | ICD10CM | Fatty (change of) liver, not elsewhere classified |
|  | K75.81 | ICD10CM | Non-alcoholic steatohepatitis (NASH) |
| MBS | 0DB64Z3 | ICD10PCS | Excision of Stomach, Percutaneous Endoscopic Approach, Vertical |
|  | 0D1647A | ICD10PCS | Bypass Stomach to Jejunum with Autologous Tissue Substitute, Percutaneous Endoscopic Approach |
|  | 0D164ZA | ICD10PCS | Bypass Stomach to Jejunum, Percutaneous Endoscopic Approach |
|  | 0D1607A | ICD10PCS | Bypass Stomach to Jejunum with Autologous Tissue Substitute, Open Approach |
|  | 0D160ZA | ICD10PCS | Bypass Stomach to Jejunum, Open Approach |
|  | 0DB60Z3 | ICD10PCS | Excision of Stomach, Open Approach, Vertical |
|  | 0DB64Z3 | ICD10PCS | Excision of Stomach, Percutaneous Endoscopic Approach, Vertical |
|  | 0DB63Z3 | ICD10PCS | Excision of Stomach, Percutaneous Approach, Vertical |
|  | 43775 | CPT | Laparoscopy, surgical, gastric restrictive procedure; longitudinal gastrectomy (ie, sleeve gastrectomy) |
|  | 87604009 | SNOMED | Sleeve resection of stomach |
|  | 427074001 | SNOMED | Laparoscopic sleeve gastrectomy |
|  | 43645 | CPT | Laparoscopy, surgical, gastric restrictive procedure; with gastric bypass and small intestine reconstruction to limit absorption |
|  | 43847 | CPT | Gastric restrictive procedure, with gastric bypass for morbid obesity; with small intestine reconstruction to limit absorption |
|  | 43846 | CPT | Gastric restrictive procedure, with gastric bypass for morbid obesity; with short limb (150 cm or less) Roux-en-Y gastroenterostomy |
|  | 43644 | CPT | Laparoscopy, surgical, gastric restrictive procedure; with gastric bypass and Roux-en-Y gastroenterostomy (roux limb 150 cm or less) |
|  | 1014146 | CPT | Gastric restrictive procedure, with gastric bypass for morbid obesity |
| Semaglutide | 1991302 | RxNORM | Semaglutide |

**Table S2** Exclusion criteria in this study.

| **Category** | **Code** | **Code Type** | **Description** |
| --- | --- | --- | --- |
| Weight loss medications | 37925 | RxNORM | orlistat |
|  | 38404, 8152 | RxNORM | topiramate AND phentermine |
|  | 42347, 7243 | RxNORM | bupropion AND naltrexone |
|  | 475968 | RxNORM | liraglutide |
|  | 2601723 | RxNORM | tirzepatide |
|  | 2469247 | RxNORM | setmelanotide |
| Types of MBS | 0DV64CZ | ICD10PCS | Restriction of Stomach with Extraluminal Device, Percutaneous Endoscopic Approach |
|  | 0DQ64ZZ | ICD10PCS | Repair Stomach, Percutaneous Endoscopic Approach |
|  | Z46.51 | ICD10CM | Encounter for fitting & adjustment of gastric lap band |
|  | 43770 | CPT | Laparoscopy, surgical, gastric restrictive procedure; placement of adjustable gastric restrictive device (e.g., gastric band and subcutaneous port components) |
|  | 43845 | CPT | Gastric restrictive procedure with partial gastrectomy, pylorus-preserving duodenoileostomy and ileoileostomy (50 to 100 cm common channel) to limit absorption (biliopancreatic diversion with duodenal switch) |
|  | 43842 | CPT | Gastric restrictive procedure, without gastric bypass, for morbid obesity; vertical-banded gastroplasty |
|  | 414574004 | SNOMED | Laparoscopic adjustable gastric banding |
| MBS generic (used only in Semaglutide cohort) | 43071500 | SNOMED | Bariatric operative procedure |
|  | Z98.84 | ICD10CM | Bariatric surgery status |
|  | 1007385 | CPT | Bariatric Surgery procedures |
| Causes of chronic liver disease | K70 | ICD10CM | Alcoholic liver disease |
|  | K70.3 | ICD10CM | Alcoholic cirrhosis of liver |
|  | K71 | ICD10CM | Toxic liver disease |
|  | K74.3 | ICD10CM | Primary biliary cirrhosis |
|  | K74.4 | ICD10CM | Secondary biliary cirrhosis |
|  | K74.5 | ICD10CM | Biliary cirrhosis, unspecified |
|  | K75.4 | ICD10CM | Autoimmune hepatitis |
|  | K76.5 | ICD10CM | Hepatic veno-occlusive disease |
|  | K83.01 | ICD10CM | Primary sclerosing cholangitis |
|  | B15-B19 | ICD10CM | Viral hepatitis |
|  | I82.0 | ICD10CM | Budd-Chiari syndrome |
|  | E88.01 | ICD10CM | Alpha-1-antitrypsin deficiency |
|  | E83.01 | ICD10CM | Wilson's disease |
|  | E83.11 | ICD10CM | Hemochromatosis |
| Other criteria with time constraint: occurred before index event | 1007811 | CPT | Liver Transplantation Procedures |
|  | Z94.4 | ICD10CM | Liver Transplant Status |
|  | 47135 | CPT | Liver allotransplantation, orthotopic, partial or whole, from cadaver or living donor, any age |
|  | 18027006 | SNOMED | Transplantation of liver |
|  | 17442500 | SNOMED | Orthotopic liver transplant |
|  | F10 | ICD10CM | Alcohol related disorders |
|  | C16 | ICD10CM | Malignant neoplasm of stomach |
|  | n/a | n/a | Deceased |

**Table S3** Study clinical outcomes with associated codes.

| **Category** | **Code** | **Code Type** | **Description** |
| --- | --- | --- | --- |
| MALO | I85.01 | ICD10CM | Oesophageal varices with bleeding |
|  | I85.11 | ICD10CM | Secondary oesophageal varices with bleeding |
|  | K72 | ICD10CM | Hepatic failure, not elsewhere classified |
|  | K76.82 | ICD10CM | Hepatic encephalopathy |
|  | R18 | ICD10CM | Ascites |
|  | K76.7 | ICD10CM | Hepatorenal syndrome |
|  | K76.81 | ICD10CM | Hepatopulmonary syndrome |
|  | Z94.4 | ICD10CM | Liver transplant status |
|  | 1007811 | CPT | Liver Transplantation Procedures |
|  | 47135 | CPT | Liver allotransplantation, orthotopic, partial or whole |
|  | 174425003 | SNOMED | Orthotopic liver transplant |
|  | 18027006 | SNOMED | Transplantation of liver |
| Cirrhosis | K74.6 | ICD10CM | Other and unspecified cirrhosis of liver |
| MACE | I21 | ICD10CM | Acute myocardial infarction |
|  | I61 | ICD10CM | Nontraumatic intracerebral haemorrhage |
|  | I63 | ICD10CM | Cerebral infarction |
| Heart failure | I50 | ICD10CM | Heart failure |
|  | I11.0 | ICD10CM | Hypertensive heart disease with heart failure |
| OAC | C18 | ICD10CM | Malignant neoplasm of colon |
|  | C25 | ICD10CM | Malignant neoplasm of pancreas |
|  | C20 | ICD10CM | Malignant neoplasm of rectum |
|  | C23 | ICD10CM | Malignant neoplasm of gallbladder |
|  | C50 | ICD10CM | Malignant neoplasm of breast |
|  | C54.1 | ICD10CM | Malignant neoplasm of endometrium |
|  | C15 | ICD10CM | Malignant neoplasm of oesophagus |
|  | C64 | ICD10CM | Malignant neoplasm of kidney, except renal pelvis |
|  | C56 | ICD10CM | Malignant neoplasm of ovary |
|  | C19 | ICD10CM | Malignant neoplasm of rectosigmoid junction |
|  | C90.0 | ICD10CM | Multiple myeloma |
|  | C70 | ICD10CM | Malignant neoplasm of meninges |
|  | C73 | ICD10CM | Malignant neoplasm of thyroid gland |
|  | C16 | ICD10CM | Malignant neoplasm of stomach |
|  | C22.0 | ICD10CM | Liver cell carcinoma |
| ACM | n/a | n/a | Deceased |

**Table S4** Variables included in propensity score matching in this study.

| **Code** | **Code Type** | **Characteristic** |
| --- | --- | --- |
| n/a | | Age at index event |
| n/a | | Female sex |
| n/a | | White |
| n/a | | Black or African American |
| n/a | | Asian |
| n/a | | Hispanic or Latino |
| 9083 | TNX Curated | Body mass index |
| 9037 | TNX Curated | Haemoglobin A1c total in Blood |
| E78 | ICD10CM | Disorders of lipoprotein metabolism and other lipidaemias |
| F17 | ICD10CM | Nicotine dependence |
| I10-I1A | ICD10CM | Hypertensive diseases |
| I20-I25 | ICD10CM | Ischemic heart diseases |
| K74.0 | ICD10CM | Hepatic fibrosis |
| K74.6 | ICD10CM | Other and unspecified cirrhosis of liver |
| 6809 | TNX Curated | Metformin |
| HS501 | VA | Insulin |

**Table S5** Baseline characteristics of patients with co-existing MASLD and T2D, with a history of sleeve gastrectomy or semaglutide use during 2018-2023.

| **Characteristic** | **Before PSM** | | | **After PSM** | | |
| --- | --- | --- | --- | --- | --- | --- |
|  | **SG n=2,507** | **Semaglutide**  **n= 22,264** | **SMD** | **SG n=2,235** | **Semaglutide n=2,235** | **SMD** |
| Age, mean [SD], years | 46 [12] | 58 [13] | 0.957 | 47 [12] | 47 [14] | 0.002 |
| Female sex, No. (%) | 1,716 (68.4) | 13,084 (58.8) | 0.202 | 1,513 (67.7) | 1,475 (66.0) | 0.036 |
| **Race & Ethnicity, n (%)** | | | | | | |
| Asian | 27 (1.1) | 1,297 (5.8) | 0.262 | 27 (1.2) | 31 (1.4) | 0.016 |
| Black or African American | 438 (17.5) | 2,483 (11.2) | 0.181 | 372 (16.6) | 351 (15.7) | 0.026 |
| Hispanic or Latino | 360 (14.4) | 1,836 (8.2) | 0.194 | 313 (14.0) | 309 (13.8) | 0.005 |
| White | 1,459 (58.2) | 16,402 (73.7) | 0.331 | 1,347 (60.3) | 1,348 (60.3) | 0.001 |
| **Anthropometric and biochemical measurements, mean [SD]** | | | | | | |
| BMI, kg/m^2^ | 45.7 [7.8] | 36.7 [7.6] | 1.162 | **45.0 [7.6]** | **43.6 [8.1]** | **0.176** |
| HbA_1C_, *%* | 6.9 [1.3] | 8.2 [1.9] | 0.842 | **7.0 [1.4]** | **7.1 [1.4]** | **0.104** |
| **Comorbidities, n (%)** | | | | | | |
| Hepatic Fibrosis | 21 (0.8) | 167 (0.8) | 0.010 | 21 (0.9) | 20 (0.9) | 0.005 |
| Hepatic Cirrhosis | 22 (0.9) | 573 (2.6) | 0.131 | 21 (0.9) | 19 (0.9) | 0.010 |
| Hypertension | 1,428 (57.0) | 10,045 (45.1) | 0.239 | 1,247 (55.8) | 1,325 (59.3) | 0.071 |
| Dyslipidaemia | 1,031 (41.1) | 8,930 (40.1) | 0.021 | 919 (41.1) | 948 (42.4) | 0.026 |
| IHD | 142 (5.7) | 2,224 (10.0) | 0.162 | 138 (6.2) | 153 (6.8) | 0.027 |
| Nicotine dependence | 83 (3.3) | 943 (4.2) | 0.049 | 78 (3.5) | 83 (3.7) | 0.012 |
| **Medication, n (%)** | | | | | | |
| Insulin | 153 (6.1) | 3,576 (16.1) | 0.321 | 150 (6.7) | 182 (8.1) | 0.055 |
| Metformin | 308 (12.3) | 4,814 (21.6) | 0.251 | 295 (13.2) | 314 (14.0) | 0.025 |

**Abbreviations:** BMI, body mass index; HbA_1C_, haemoglobin A_1C_**;** IHD, ischemic heart disease; n, number; PSM, propensity score matching; SG, sleeve gastrectomy; SMD, standardized mean difference; SD, standard deviation; T2D, type 2 diabetes.

**Table S6** Baseline characteristics of patients with co-existing MASLD and T2D, with a history of Roux-en-Y gastric bypass or semaglutide use during 2018-2023.

| **Characteristic** | **Before PSM** | | | **After PSM** | | |
| --- | --- | --- | --- | --- | --- | --- |
|  | **RYGB n=1,840** | **Semaglutide**  **n= 22,264** | **SMD** | **RYGB n=1,715** | **Semaglutide n=1,715** | **SMD** |
| Age, mean [SD], years | 48 [12] | 58 [13] | 0.792 | 49 [12] | 49 [14] | 0.017 |
| Female sex, No. (%) | 1,396 (75.9) | 13,084 (58.8) | 0.371 | 1,290 (75.2) | 1,287 (75.0) | 0.004 |
| **Race & Ethnicity, n (%)** | | | | | | |
| Asian | 24 (1.3) | 1,297 (5.8) | 0.246 | 24 (1.4) | 26 (1.5) | 0.010 |
| Black or African American | 204 (11.1) | 2,483 (11.2) | 0.002 | 192 (11.2) | 195 (11.4) | 0.006 |
| Hispanic or Latino | 418 (22.7) | 1,836 (8.2) | 0.408 | 366 (21.3) | 340 (19.8) | 0.038 |
| White | 1,334 (72.5) | 16,402 (73.7) | 0.026 | 1,241 (72.4) | 1,223 (71.3) | 0.023 |
| **Anthropometric and biochemical measurements, mean [SD]** | | | | | | |
| BMI, kg/m^2^ | 43.9 [8.6] | 36.7 [7.6] | 0.880 | 43.4 [8.5] | 43.1 [7.8] | 0.037 |
| HbA_1C_, *%* | 6.9 [1.2] | 8.2 [1.8] | 0.827 | **7.0 [1.3]** | **7.1 [1.4]** | **0.109** |
| **Comorbidities, n (%)** | | | | | | |
| Hepatic Fibrosis | 21 (1.1) | 167 (0.8) | 0.040 | 20 (1.2) | 17 (1.0) | 0.017 |
| Hepatic Cirrhosis | 26 (1.4) | 573 (2.6) | 0.083 | 26 (1.5) | 32 (1.9) | 0.027 |
| Hypertension | 1,090 (59.2) | 10,045 (45.1) | 0.286 | 997 (58.1) | 1,034 (60.3) | 0.044 |
| Dyslipidaemia | 751 (40.8) | 8,930 (40.1) | 0.014 | 699 (40.8) | 714 (41.6) | 0.018 |
| IHD | 122 (6.6) | 2,224 (10.0) | 0.122 | 116 (6.8) | 143 (8.3) | 0.060 |
| Nicotine dependence | 78 (4.2) | 943 (4.2) | <0.001 | 74 (4.3) | 77 (4.5) | 0.009 |
| **Medication, n (%)** |  |  |  |  |  |  |
| Insulin | 181 (9.8) | 3,576 (16.1) | 0.186 | 180 (10.5) | 212 (12.4) | 0.059 |
| Metformin | 189 (10.3) | 4,814 (21.6) | 0.314 | 187 (10.9) | 189 (11.0) | 0.004 |

**Abbreviations:** BMI, body mass index; HbA_1C_, haemoglobin A_1C_**;** IHD, ischemic heart disease; n, number; PSM, propensity score matching; RYGB, Roux-en-Y gastric bypass; SMD, standardized mean difference; SD, standard deviation; T2D, type 2 diabetes.

**Table S7** Baseline characteristics of patients with MASLD and *without* cirrhosis, with a history of metabolic bariatric surgery or semaglutide use during 2018-2023.

| **Characteristic** | **Before PSM** | | | **After PSM** | | |
| --- | --- | --- | --- | --- | --- | --- |
|  | **MBS n=4,317** | **Semaglutide**  **n= 20,959** | **SMD** | **MBS n=3,387** | **Semaglutide n=3,387** | **SMD** |
| Age, mean [SD], years | 47 [12] | 57 [13] | 0.876 | 49 [11] | 48 [13] | 0.029 |
| Female sex, No. (%) | 3,101 (71.8) | 12,288 (58.6) | 0.280 | 2,348 (69.3) | 2,356 (69.6) | 0.005 |
| **Race & Ethnicity, n (%)** | | | | | | |
| Asian | 52 (1.2) | 1,248 (6.0) | 0.258 | 49 (1.4) | 52 (1.5) | 0.007 |
| Black or African American | 648 (15.0) | 2,423 (11.6) | 0.102 | 476 (14.1) | 469 (13.8) | 0.006 |
| Hispanic or Latino | 774 (17.9) | 1,723 (8.2) | 0.291 | 520 (15.4) | 535 (15.8) | 0.012 |
| White | 2,756 (63.8) | 15,307 (73.0) | 0.199 | 2,242 (66.2) | 2,229 (65.8) | 0.008 |
| **Anthropometric and biochemical measurements, mean [SD]** | | | | | | |
| BMI, kg/m^2^ | 44.8 [8.2] | 36.7 [7.6] | 1.020 | 43.5 [8.0] | 42.7 [7.8] | 0.097 |
| HbA_1C_, *%* | 6.9 [1.3] | 8.2 [1.8] | 0.844 | **7.1 [1.3]** | **7.2 [1.5]** | **0.116** |
| **Comorbidities, n (%)** | | | | | | |
| Hepatic Fibrosis | 30 (0.7) | 90 (0.4) | 0.040 | 23 (0.7) | 22 (0.6) | 0.004 |
| Hypertension | 2,488 (57.6) | 9,444 (45.1) | 0.254 | 1,874 (55.3) | 1,902 (56.2) | 0.017 |
| Dyslipidaemia | 1,763 (40.8) | 8,459 (40.4) | 0.010 | 1,401 (41.4) | 1,401 (41.4) | <0.001 |
| IHD | 260 (6.0) | 2,061 (9.8) | 0.141 | 237 (7.0) | 238 (7.0) | 0.001 |
| Nicotine dependence | 160 (3.7) | 898 (4.3) | 0.030 | 135 (4.0) | 138 (4.1) | 0.005 |
| **Medication, n (%)** |  |  |  |  |  |  |
| Insulin | 323 (7.5) | 3,300 (15.7) | 0.260 | 297 (8.8) | 345 (10.2) | 0.048 |
| Metformin | 486 (11.3) | 4,595 (21.9) | 0.290 | 428 (12.6) | 430 (12.7) | 0.002 |

**Abbreviations:** BMI, body mass index; HbA_1C_, haemoglobin A_1C_**;** IHD, ischemic heart disease; MBS, metabolic bariatric surgery; n, number; PSM, propensity score matching; SMD, standardized mean difference; SD, standard deviation; T2D, type 2 diabetes.

**Table S8** Baseline characteristics of patients with MASLD and *without* T2D, with a history of metabolic bariatric surgery or semaglutide use during 2021-2023.

| **Characteristic** | **Before PSM** | | | **After PSM** | | |
| --- | --- | --- | --- | --- | --- | --- |
|  | **MBS n=3,303** | **Semaglutide**  **n= 5,847** | **SMD** | **MBS n=2,237** | **Semaglutide n=2,237** | **SMD** |
| Age, mean [SD], years | 41 [11] | 49 [14] | 0.677 | 43 [11] | 43 [13] | 0.028 |
| Female sex, No. (%) | 2,658 (80.5) | 4,013 (68.6) | 0.274 | 1,725 (77.1) | 1,741 (77.8) | 0.017 |
| **Race & Ethnicity, No. (%)** | | | | | | |
| Asian | 32 (1.0) | 161 (2.8) | 0.132 | 29 (1.3) | 28 (1.3) | 0.004 |
| Black or African American | 575 (17.4) | 406 (6.9) | 0.324 | 273 (12.2) | 274 (12.2) | 0.001 |
| White | 2,062 (62.4) | 4,651 (79.5) | 0.384 | 1,566 (70.0) | 1,549 (69.2) | 0.017 |
| Hispanic or Latino | 522 (15.8) | 449 (7.7) | 0.254 | 272 (12.2) | 265 (11.8) | 0.010 |
| **Anthropometric and biochemical measurements, mean [SD]** | | | | | | |
| BMI, kg/m^2^ | 45.0 [7.4] | 38.9 [7.6] | 0.809 | 43.5 [7.4] | 42.9 [7.4] | 0.092 |
| **Comorbidities, No. (%)** | | | | | | |
| Hepatic Fibrosis | 17 (0.5) | 51 (0.9) | 0.043 | 15 (0.7) | 14 (0.6) | 0.006 |
| Hepatic Cirrhosis | 11 (0.3) | 39 (0.7) | 0.047 | 10 (0.4) | 10 (0.4) | <0.001 |
| Hypertension | 993 (30.1) | 1,422 (24.3) | 0.129 | 646 (28.9) | 665 (29.7) | 0.019 |
| Dyslipidaemia | 549 (16.6) | 1,217 (20.8) | 0.108 | 407 (18.2) | 423 (18.9) | 0.018 |
| IHD | 37 (1.1) | 179 (3.1) | 0.136 | 36 (1.6) | 35 (1.6) | 0.004 |
| Nicotine dependence | 78 (2.4) | 135 (2.3) | 0.004 | 50 (2.2) | 52 (2.3) | 0.006 |

**Abbreviations:** BMI, body mass index; IHD, ischemic heart disease; n, number; MBS, metabolic bariatric surgery; PSM, propensity score matching; SMD, standardized mean difference; SD, standard deviation; T2D, type 2 diabetes.

**Table S9** Major adverse hepatic and extrahepatic clinical outcomes and all-cause mortality in individuals with MASLD and T2D, having had MBS or semaglutide between 2018-2023; *Sensitivity analysis with follow-up window starting 90 days after the index event.*

| **Outcome** | **Patients in MBS cohort, n** | **Patients with outcome in MBS cohort, n (%)** | **Patients in semaglutide cohort, n** | **Patients with outcome in semaglutide cohort, n (%)** | **Hazard Ratio (CI)** | **p-value for hazard proportionality test**^†^ |
| --- | --- | --- | --- | --- | --- | --- |
| **Hepatic outcomes** | | | | | |  |
| MALO *(any)^†^* | 3,522 | 66 (1.9) | 3,522 | 46 (1.3) | 1.59  (1.09 – 2.32) | 0.368 |
| Cirrhosis *(first)* ^††^ | 3,383 | 27 (0.8) | 3,375 | 54 (1.6) | 0.55  (0.35 – 0.88) | 0.859 |
| **Extrahepatic outcomes** | | | | | |  |
| MACE *(any)*^†^ | 3,522 | 75 (2.1) | 3,522 | 154 (4.4) | 0.53  (0.40 – 0.70) | 0.341 |
| Heart failure *(first)* ^††^ | 3,213 | 32 (1.0) | 2,998 | 75 (2.5) | 0.44  (0.29 – 0.67) | 0.217 |
| Obesity-associated cancer *(first)*^††^ | 3,317 | 24 (0.7) | 3,228 | 48 (1.5) | 0.54  (0.33 – 0.88) | 0.734 |
| **All-cause mortality** | | | | | |  |
| Deceased | 3,522 | 50 (1.4) | 3,522 | 51 (1.4) | 1.09  (0.74 – 1.61) | 0.379 |

**Abbreviations:** CI, confidence interval; MACE, major adverse cardiovascular events; MALO, major adverse liver outcomes; MBS, metabolic bariatric surgery; n, number; Obesity-associated cancer is defined as cancer of any of the following: colon, sigmoid, rectum, pancreas, gallbladder, stomach, oesophagus, hepatocellular carcinoma, breast, endometrium, ovary, kidney, thyroid, meninges and multiple myeloma.

† In hazards proportionality test, if p value is >0.05, the hazard ratio remains constant over time

**Table S10** Major adverse hepatic and extrahepatic clinical outcomes and
all-cause mortality in individuals with MASLD and *without* cirrhosis, having had MBS or semaglutide between 2018-2023.

| **Outcome** | **Patients in MBS cohort, n** | **Patients with outcome in MBS cohort, n (%)** | **Patients in semaglutide cohort, n** | **Patients with outcome in semaglutide cohort, n (%)** | **Hazard Ratio (CI)** | **p-value for hazard proportionality test**^†^ |
| --- | --- | --- | --- | --- | --- | --- |
| **Hepatic outcomes** | | | | | |  |
| MALO *(any)^†^* | 3,387 | 55 (1.6) | 3,387 | 36 (1.1) | 1.69  (1.11– 2.57) | 0.100 |
| Cirrhosis *(first)* ^††^ | 3,369 | 23 (0.7) | 3,381 | 52 (1.5) | 0.49  (0.30 – 0.80) | 0.692 |
| **Extrahepatic outcomes** | | | | | |  |
| MACE *(any)*^†^ | 3,387 | 68 (2.0) | 3,387 | 152 (4.5) | 0.48  (0.36 – 0.65) | 0.563 |
| Heart failure *(first)* ^††^ | 3,099 | 29 (0.9) | 2,947 | 81 (2.7) | 0.38  (0.25 – 0.58) | 0.273 |
| Obesity-associated cancer *(first)*^††^ | 3,203 | 22 (0.7) | 3,118 | 40 (1.3) | 0.59  (0.35 – 0.99) | 0.138 |
| **All-cause mortality** | | | | | |  |
| Deceased | 3,387 | 44 (1.3) | 3,387 | 49 (1.4) | 0.99  (0.66 – 1.49) | 0.739 |

**Abbreviations:** CI, confidence interval; MACE, major adverse cardiovascular events; MALO, major adverse liver outcomes; MBS, metabolic bariatric surgery; n, number; Obesity-associated cancer is defined as cancer of any of the following: colon, sigmoid, rectum, pancreas, gallbladder, stomach, oesophagus, hepatocellular carcinoma, breast, endometrium, ovary, kidney, thyroid, meninges and multiple myeloma.

† In hazards proportionality test, if p value is >0.05, the hazard ratio remains constant over time

**Table S11** Major adverse hepatic and extrahepatic clinical outcomes and all-cause mortality in individuals with co-existing MASLD and T2D, having had Roux-en-Y gastric bypass or semaglutide between 2018-2023.

| **Outcome** | **Patients in MBS cohort, n** | **Patients with outcome in MBS cohort, n (%)** | **Patients in semaglutide cohort, n** | **Patients with outcome in semaglutide cohort, n (%)** | **Hazard Ratio (CI)** | **p-value for hazard proportionality test**^†††^ |
| --- | --- | --- | --- | --- | --- | --- |
| **Hepatic outcomes** | | | | | |  |
| MALO *(any)^†^* | 1,715 | 51 (3.0) | 1,715 | 27 (1.6) | 2.01  (1.26-3.20) | 0.738 |
| MALO *(first)* ^††^ | 1,621 | 35 (2.2) | 1,650 | 16 (1.0) | 2.35  (1.30-4.25) | 0.239 |
| Cirrhosis *(first)* ^††^ | 1,634 | 18 (1.1) | 1,638 | 31 (1.9) | 0.61  (0.34 – 1.09) | 0.362 |
| **Extrahepatic outcomes** | | | | | |  |
| MACE *(any)*^†^ | 1,715 | 37 (2.2) | 1,715 | 91 (5.3) | 0.42  (0.29 -0.62) | 0.807 |
| MACE *(first)* ^††^ | 1,629 | 25 (1.5) | 1,553 | 47 (3.0) | 0.53  (0.33 – 0.87) | 0.795 |
| Heart failure *(first)* ^††^ | 1,571 | 21 (1.3) | 1,457 | 42 (2.9) | 0.48  (0.29-0.82) | 0.175 |
| Obesity-associated cancer *(first)*^††^ | 1,594 | 19 (1.2) | 1,569 | 18 (1.1) | 1.09  (0.57-2.07) | 0.913 |
| **All-cause mortality** | | | | | |  |
| Deceased | 1,715 | 42 (2.4) | 1,715 | 27 (1.6) | 1.64  (1.01– 2.67) | 0.711 |

**Abbreviations:** CI, confidence interval; MACE, major adverse cardiovascular events; MALO, major adverse liver outcomes; MBS, metabolic bariatric surgery; n, number; Obesity-associated cancer is defined as cancer of any of the following: colon, sigmoid, rectum, pancreas, gallbladder, stomach, oesophagus, hepatocellular carcinoma, breast, endometrium, ovary, kidney, thyroid, meninges and multiple myeloma.

† any diagnosis includes outcomes recorded during the study follow-up that are either first ever occurrence or a recurrence, i.e. the outcome could have occurred in the past before the subject recruitment into the study or before the start of follow-up

†† first diagnosis signifies first ever occurrence only, i.e. no previous history of a given outcome before the subject recruitment into the study and start of follow-up

††† In hazards proportionality test, if p value is >0.05, the hazard ratio remains constant over time

**Table S12** Major adverse hepatic and extrahepatic clinical outcomes and all-cause mortality in individuals with co-existing MASLD and T2D, having had sleeve gastrectomy or semaglutide between 2018-2023.

| **Outcome** | **Patients in MBS cohort, n** | **Patients with outcome in MBS cohort, n (%)** | **Patients in semaglutide cohort, n** | **Patients with outcome in semaglutide cohort, n (%)** | **Hazard Ratio (CI)** | **p-value for hazard proportionality test**^††††^ |
| --- | --- | --- | --- | --- | --- | --- |
| **Hepatic outcomes** | | | | | |  |
| MALO *(any)^†^* | 2,235 | 19 (0.9) | 2,235 | 22 (1.0) | 0.97  (0.52-1.78) | 0.614 |
| MALO *(first)* ^††^ | 2,199 | 12 (0.5) | 2,150 | 11 (0.5) | 1.21  (0.53-2.74) | 0.704 |
| Cirrhosis *(first)* ^††^ | n/a | n/a | n/a | n/a | n/a | n/a |
| **Extrahepatic outcomes** | | | | | |  |
| MACE *(any)*^†^ | 2,235 | 37 (1.7) | 2,235 | 94 (4.2) | 0.43  (0.30 -0.63) | 0.995 |
| MACE *(first)* ^††^ | 2,112 | 13 (0.6) | 2,061 | 46 (2.2) | 0.31  (0.17 – 0.58) | 0.778 |
| Heart failure *(first)* ^††^ | 2,036 | ≤10^†††^ (0.5) | 1,929 | 46 (2.4) | 0.24  (0.12-0.47) | 0.601 |
| Obesity-associated cancer *(first)*^††^ | n/a | n/a | n/a | n/a | n/a | n/a |
| **All-cause mortality** | | | | | |  |
| Deceased | 2,235 | 12 (0.5) | 2,235 | 31 (1.4) | 0.44  (0.23 – 0.85) | 0.813 |

**Abbreviations:** CI, confidence interval; MACE, major adverse cardiovascular events; MALO, major adverse liver outcomes; MBS, metabolic bariatric surgery; n, number; Obesity-associated cancer is defined as cancer of any of the following: colon, sigmoid, rectum, pancreas, gallbladder, stomach, oesophagus, hepatocellular carcinoma, breast, endometrium, ovary, kidney, thyroid, meninges and multiple myeloma.

† any diagnosis includes outcomes recorded during the study follow-up that are either first ever occurrence or a recurrence, i.e. the outcome could have occurred in the past before the subject recruitment into the study or before the start of follow-up

†† first diagnosis signifies first ever occurrence only, i.e. no previous history of a given outcome before the subject recruitment into the study and start of follow-up

††† to protect patient privacy, numbers are rounded up to 10 within TriNetX. This might impact results, particularly for small cohorts and infrequent outcomes.

†††† In hazards proportionality test, if p value is >0.05, the hazard ratio remains constant over time

**Table S13** Major adverse hepatic and extrahepatic clinical outcomes and all-cause mortality in individuals with MASLD and without T2D, having had MBS or semaglutide between 2021-2023.

| **Outcome** | **Patients in MBS cohort, n** | **Patients with outcome in MBS cohort, n (%)** | **Patients in semaglutide cohort, n** | **Patients with outcome in semaglutide cohort, n (%)** | **Hazard Ratio (CI)** | **p-value for hazard proportionality test**^††††^ |
| --- | --- | --- | --- | --- | --- | --- |
| **Hepatic outcomes** | | | | | |  |
| MALO *(any)^†^* | 2,237 | 17 (0.8) | 2,237 | 14 (0.6) | 1.47  (0.73-2.98) | 0.097 |
| MALO *(first)* ^††^ | 2,222 | 14 (0.6) | 2,217 | 12 (0.5) | 1.42  (0.66-3.07) | 0.008 |
| Cirrhosis *(first)* ^††^ | n/a | n/a | n/a | n/a | n/a | n/a |
| **Extrahepatic outcomes** | | | | | |  |
| MACE *(any)*^†^ | n/a | n/a | n/a | n/a | n/a | n/a |
| MACE *(first)* ^††^ | n/a | n/a | n/a | n/a | n/a | n/a |
| Heart failure *(first)* ^††^ | n/a | n/a | n/a | n/a | n/a | n/a |
| Obesity- associated cancer *(first)*^††^ | 2,174 | ≤10^†††^ (0.5) | 2,113 | 20 (0.9) | 0.60  (0.28 – 1.28) | 0.409 |
| **All-cause mortality** | | | | | |  |
| Deceased | n/a | n/a | n/a | n/a | n/a | n/a |

**Abbreviations:** CI, confidence interval; MACE, major adverse cardiovascular events; MALO, major adverse liver outcomes; MBS, metabolic bariatric surgery; n, number; n/a, analysis not performed because the number of events in both cohorts was too low; Obesity-associated cancer is defined as cancer of any of the following: colon, sigmoid, rectum, pancreas, gallbladder, stomach, oesophagus, hepatocellular carcinoma, breast, endometrium, ovary, kidney, thyroid, meninges and multiple myeloma.

† any diagnosis includes outcomes recorded during the study follow-up that are either first ever occurrence or a recurrence, i.e. the outcome could have occurred in the past before the subject recruitment into the study or before the start of follow-up

†† first diagnosis signifies first ever occurrence only, i.e. no previous history of a given outcome before the subject recruitment into the study and start of follow-up

††† to protect patient privacy, numbers are rounded up to 10 within TriNetX. This might impact results, particularly for small cohorts and infrequent outcomes.

†††† In hazards proportionality test, if p value is >0.05, the hazard ratio remains constant over time
